# Mach-Zehnder Interferometer Biochemical Sensor Based on Silicon-on-Insulator Rib Waveguide with Large Cross Section

**DOI:** 10.3390/s150921500

**Published:** 2015-08-28

**Authors:** Dengpeng Yuan, Ying Dong, Yujin Liu, Tianjian Li

**Affiliations:** 1 Graduate School at Shenzhen, Tsinghua University, J209A, Tsinghua Campus, University Town of Shenzhen, Shenzhen 518055, China; E-Mails: ydp12@mails.tsinghua.edu.cn (D.Y.); liuyj13@mails.tsinghua.edu.cn (Y.L.); litj13@mails.tsinghua.edu.cn (T.L.); 2 Institute of Material, China Academy of Engineering Physics, Jiangyou 621908, China

**Keywords:** Mach-Zehnder interferometer (MZI), Silicon-on-insulator (SOI), biochemical sensor, rib waveguide, finite difference-time domain (FDTD) method

## Abstract

A high-sensitivity Mach-Zehnder interferometer (MZI) biochemical sensing platform based on Silicon-in-insulator (SOI) rib waveguide with large cross section is proposed in this paper. Based on the analyses of the evanescent field intensity, the mode polarization and cross section dimensions of the SOI rib waveguide are optimized through finite difference method (FDM) simulation. To realize high-resolution MZI read-out configuration based on the SOI rib waveguide, medium-filled trenches are employed and their performances are simulated through two-dimensional finite-difference-time domain (2D-FDTD) method. With the fundamental EH-polarized mode of the SOI rib waveguide with a total rib height of 10 μm, an outside rib height of 5 μm and a rib width of 2.5 μm at the operating wavelength of 1550 nm, when the length of the sensitive window in the MZI configuration is 10 mm, a homogeneous sensitivity of 7296.6%/refractive index unit (RIU) is obtained. Supposing the resolutions of the photoelectric detectors connected to the output ports are 0.2%, the MZI sensor can achieve a detection limit of 2.74 × 10^−6^ RIU. Due to high coupling efficiency of SOI rib waveguide with large cross section with standard single-mode glass optical fiber, the proposed MZI sensing platform can be conveniently integrated with optical fiber communication systems and (opto-) electronic systems, and therefore has the potential to realize remote sensing, *in situ* real-time detecting, and possible applications in the internet of things.

## 1. Introduction

With the capability of efficiently transforming chemical or biological reaction into measurable signal, biochemical sensors are widely used in chemical industry, biological process, medical diagnostics, healthcare, environmental monitoring, military defense, and scientific research. Compared with other transduction methods, optical sensors possess higher sensitivity and immunity to electromagnetic interference [1]. Among them, sensors based on optical waveguide are considered as the most valuable in real-time in-situ applications due to their advantages of miniaturization and integration.

Microring resonator (MRR) [2,3,4] and Mach-Zehnder interferometer (MZI) [5,6] are the two most common configurations employed in waveguide-based sensors for optical signal read-out. MRR biochemical sensors have demonstrated high sensitivity detection of protein, DNA, virus, and bacteria [7]. In order to perform wavelength scanning, a wavelength-tunable laser and an optical spectrum analyzer are required, and the detection limit of the MRR-based sensor is ultimately restricted by the resolutions of this expensive and complex external equipment. By contrast, the MZI read-out configuration is based on the detection of optical intensity and neither tunable laser nor spectrum analyzer are required. Therefore optical waveguide MZI as a sensing platform is more feasible for the miniaturization and integration of the whole sensing system [8].

Silicon-on-insulator (SOI) is a promising material to realize a low-loss optical waveguide, and it has an extremely high refractive index contrast. SOI-based nanowire waveguide [9,10] and slot waveguide [11,12] have been employed to realize high-sensitivity biochemical sensors. The cross-sectional dimensions of these waveguides are generally hundreds of nanometers or even smaller, thus the input/output coupling parts are relatively complex, such as a grating coupler, prism coupler, or large mode converter, which results in high cost and low flexibility of the total sensing system. An SOI rib waveguide with large cross section, *i.e*. several microns in transversal dimension, possesses high coupling efficiency with standard single-mode glass fibers, and therefore has the potential for integration with optical fiber communication systems and opto-electronic systems [13].

This article proposes an MZI biochemical sensor based on an SOI rib waveguide with a large cross section. Following the introduction to the background of the research, the operation principle of the MZI sensor is presented in Section 2, then the section dimensions of an SOI rib waveguide with a large cross section are optimized based on the simulations of evanescent field sensing in Section 3. In Section 4, the implementations of the MZI structure are analyzed and discussed, including comparison with conventional approaches, numerical simulations of the trench-based waveguide bends and branches, and the performances of the proposed MZI structure. The configuration of the MZI-based sensing platform is proposed in Section 5, and the discussion and results of this research are given in the last two sections.

## 2. Principle of Operation

**Figure 1:**
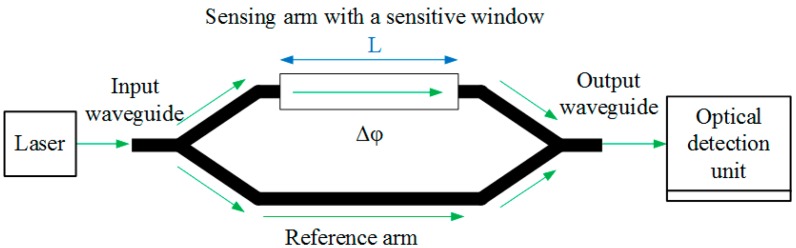
The schematic diagram of a typical Mach-Zehnder interferometer (MZI) sensor based on optical waveguide.

A typical MZI sensor based on optical waveguide consists of a laser source, an optical detection unit and an MZI structure with an evanescent field sensing window, as shown in Figure 1. The MZI structure consists of an input waveguide and an output waveguide, a beam splitter (the left Y-junction), and a beam combiner (the right Y-junction), as well as two straight waveguides between the two Y-junctions as the sensing arm (with a sensitive window) and the reference arm respectively. In the operation, the monochromatic and polarized light from the laser source is coupled into the input waveguide and split equally at the beam splitter. Then the two guiding modes propagate a certain distance along the sensing arm and the reference arm respectively, and recombine at the beam combiner. Based on evanescent field sensing, a phase difference *Δφ* between the sensing arm and the reference arm occurs when the effective refractive index of the guiding mode in the sensing arm is changed by the biochemical reaction in the sensitive window, resulting in an intensity modulation caused by the interference of the two arms at the waveguide output. Measuring the interference intensity at the output waveguide, the biochemical reaction in the sensitive window is able to be detected.

The phase difference *Δφ* can be expressed as

(1) $$\Delta \varphi =\frac{2\pi }{\lambda }\Delta N_{eff}L$$

where, *λ* represents the operating wavelength, *N_eff_* represents the effective refractive index of propagating mode of waveguide, *L* is the length of the sensitive window on the sensing arm. Assuming the detection target is the refractive index change of bulk solution, the sensitivity of MZI-based sensor can be expressed as:

(2) $$S=\frac{\Delta P}{\Delta n}$$

where, *Δn* represents the refractive index change of the sensitive region, and *ΔP* is the normalized output power change of the MZI sensor responding to a given *Δn*. As the partial sensitivity of the interference measurement and evanescent field sensing are defined as *ΔP/Δφ* and *ΔN_eff_/Δn* respectively, the Equation (2) can be rewritten as:

(3) $$S=\frac{\Delta P}{\Delta \varphi }\cdot \frac{\Delta \varphi }{\Delta N_{eff}}\cdot \frac{\Delta N_{eff}}{\Delta n}=\frac{2\pi }{\lambda }\cdot \frac{\Delta P}{\Delta \varphi }\cdot \frac{\Delta N_{eff}}{\Delta n}\cdot L$$

It can be found that the sensitivity of the MZI-based sensor is determined by the length of the sensitive window (*L*), and the partial sensitivity of the interference measurement and evanescent field sensing.

## 3. Section Dimensions of SOI Rib Waveguide

### 3.1. Intensity of Evanescent Field

**Figure 2:**
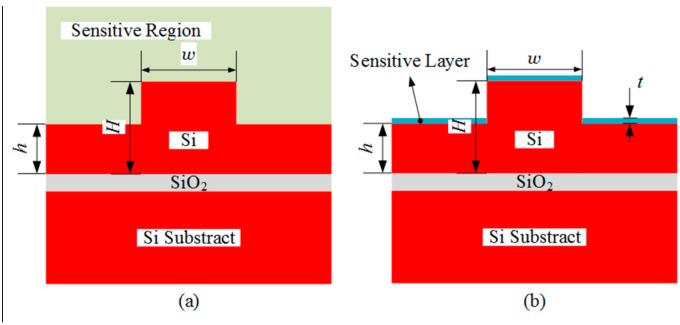
The schematic diagram of sensitive region and sensitive layer at silicon-on-insulator (SOI) rib waveguide with large cross section. (**a**) Homogeneous sensing, the light olive region is the sensitive region; (**b**) Surface sensing, the blue region represents the sensitive layer, and its thickness is denoted by *t*.

The fundamental principle of optical waveguide MZI sensor is based on the evanescent field sensing. According to the Goos-Hanchen effects, the evanescent field is a fraction of optical field that extends to the cladding layer and the substrate layer of the waveguide. In general, there are two types of evanescent field sensing, homogeneous sensing, and surface sensing [14]. The homogeneous sensing and surface sensing for SOI waveguide with large cross section are shown in Figure 2a,b respectively. For the homogeneous sensing, the effective index variation of the propagating mode is produced by the change of the refractive index in the sensitive region. While for the surface sensing, the effective index variation is produced by the change of the thickness of the ultra-thin sensitive layer which is immobilized on the waveguide surface. The thickness of the surface sensitive layer is denoted as *t* (shown in Figure 2b), which is changed at a small range, about several nanometers. Homogeneous sensing is generally used to detect the concentration change of gas or liquid in the entire sensitive region, and the surface sensing is often applied to detect protein, DNA, virus, and bacteria with the help of immobilized receptor molecules [15].

Obviously, the greater the intensity of the evanescent field is, the more sensitive the sensor both in the case of homogeneous sensing and surface sensing will be. The intensity of the evanescent field can be represented by the confinement factor *Γ_s_*, which is the ratio of the electric field intensity in the sensitive region or the sensitive layer to the entire mode distribution of the guiding mode [15], defined as Equation (4).

(4) $$\Gamma_{s}=\frac{\iint_{s}{\left|E\left(x,y\right)\right|}^{2}dxdy}{\iint_{\infty }{\left|E\left(x,y\right)\right|}^{2}dxdy}$$

Generally, in order to achieve low-loss propagation and avoid negative influences due to multimode transmission or cross-polarization interference, the optical waveguides employed in the senor must possess single mode and single polarization. In this article, the SOI rib waveguides with large cross section will be confined by single mode conditions. Once the mode distributions are solved by mode solver programs, such as finite element method (FEM) and finite difference method (FDM), the intensity of the evanescent field can be calculated through area integration. Therefore, taking the maximization of the intensity of the evanescent field as the target, the optimization of waveguide section dimensions can be realized.

### 3.2. Optimization of Waveguide Section Dimensions

As an example, suppose the sensor based on SOI rib waveguide with large cross section is used to detect the concentration of glucose solution and the refractive index *n_c_* of the analyte solution is in the vicinity of 1.33. In order to guarantee high coupling efficiency with the standard single-mode fiber, the total rib height (*H*) of SOI rib waveguide is set to be 10 μm, and the operating wavelength is selected at 1550 nm so that the refractive index of silicon and SiO_2_ are 3.476 and 1.444, respectively. Considering too small a rib width (*w*) will reduce the restriction of the rib structure on the guiding mode and too big a rib width (*w*) will be difficult for system integration (such as the waveguide bends and branches analyzed in next section), the rib width (*w*) of SOI rib waveguide is set to be in the range of 2.5 μm to 10 μm.

**Figure 3:**
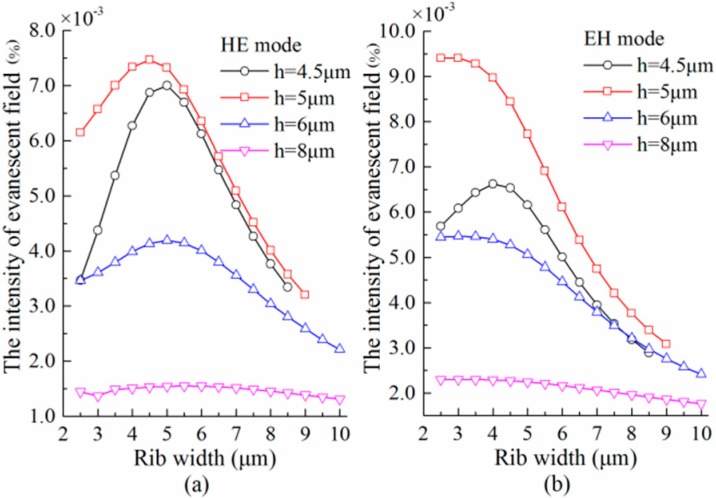
Dependence of the intensity of the evanescent field for homogeneous sensing on rib width (*w*) with different outside rib height (h). (**a**) HE polarization; (**b**) EH polarization.

The modes of rib waveguide can be denoted as HE_nm_ or EH_nm_ [16], where *n* = 0, 1, 2…, and *m* = 0, 1, 2…. HE mode and EH mode are commonly known as quasi-transverse-electric mode and quasi-transverse-magnetic mode respectively. Employing a strict single-mode condition [17], all the SOI rib waveguides with large cross section only support the fundamental guiding modes for each polarization, *i.e.* HE_00_ and EH_00_. Using the full-vector finite difference method (FDM) [18] to solve the fundamental guiding modes with different dimensions (including the outside rib height *h* and the rib width *w*), the intensity of the evanescent field for homogeneous sensing can be calculated, as shown in Figure 3.

It can be seen from Figure 3, for an SOI rib waveguide with the total rib height of 10 μm, the evanescent field intensity of homogeneous sensing exhibits a maximum of 7.436 × 10^−3^% for HE polarization, corresponding to *h* = 5 μm and *w* = 4.5 μm, and 9.407 × 10^−3^% for EH polarization, corresponding to *h* = 5 μm and *w* = 2.5 μm.

Using the same method, dependence of the evanescent field intensity for surface sensing on rib width (*w*) with different outside rib height (*h*) also can be calculated. Assuming the refractive index of the sensitive layer *n_m_* = 1.45 and the thickness of the sensitive layer *t* = 10 nm, the evanescent field intensity for surface sensing of the SOI rib waveguide with the total rib height of 10 μm possesses a maximum of 5.089 × 10^−3^% for HE polarization and 7.924 × 10^−3^% for EH polarization, both corresponding to *h* = 5 μm and *w* = 2.5 μm. Both for homogeneous sensing and surface sensing, it is evident that EH polarization of the SOI rib waveguide with large cross section is significantly more sensitive than HE polarization due to the larger evanescent field intensity.

**Figure 4:**
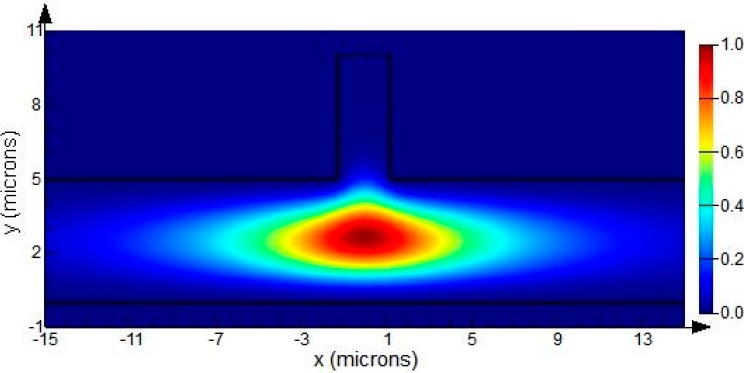
Simulation of the fundamental EH mode of the SOI rib waveguide with *H* = 10 μm, *h* = 5 μm, *w* = 2.5 μm at wavelength of 1550 nm, calculating with full-vector finite difference method (FDM).

According to the variational theorem for dielectric waveguides, an analytical method can be used to estimate the partial sensitivity of evanescent field sensing based on the calculated evanescent field intensity [17,18]. Therefore, the partial sensitivity of homogeneous sensing (*ΔN_eff_/Δn*) possesses a maximum of 2.86 × 10^−3^ for HE polarization (corresponding to *h* = 5 μm, *w* = 4.5 μm) and 3.6 × 10^−3^ for EH polarization (corresponding to *h* = 5 μm, *w* = 2.5 μm), and the partial sensitivity of surface sensing (*ΔN_eff_/Δt*) exhibits a maximum of 4.89 × 10^−6^ nm^−1^ for HE polarization and 7.61 × 10^−6^ nm^−1^ for EH polarization (both corresponding to *h* = 5μm, *w* = 2.5μm). Therefore, the result of the optimization is that the waveguide mode is the fundamental EH mode, and the cross section dimension of the SOI rib waveguide is *H* = 10 μm*, h* = 5 μm*, w* = 2.5 μm. The simulation of the optimized mode is shown in Figure 4.

## 4. MZI Structure Implementation

### 4.1. Conventional Implementations

In the waveguide MZI sensor, the evanescent field sensing is read out by the MZI configuration. The most critical components of the MZI configuration are the beam splitter and the beam combiner, which are waveguide branches and are often identical. For the conventional MZI configurations, there are three structures to realize waveguide branches, as listed in Table 1. The data in this table show the technical parameters and the minimum required length of a single branch to fulfill the separation distance of *d* = 50 μm between the two straight SOI rib waveguides with large cross section. Thus for the SOI rib waveguide with a large cross section, the conventional implementations of waveguide branches lead to an overlong structure which is difficult to be realized.

**Table 1:** The conventional implementations of waveguide branches for SOI rib waveguide with large cross section. Assuming *H* = 10 μm, *h* = 5 μm, *w* = 5 μm.

Structure Type	Technology Platform	Technical Parameters & Requirements
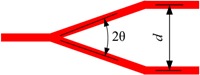 Y-junction	Mode-matching	When *d* = 50 μm, *2θ* = 0.4°, The minimum length of a single branch: *L_0_* = 7.2 mm.
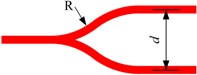 S-bend splitter	Waveguide bending	When *d* = 50 μm, *R* > 0.26 m, The minimum length of a single branch: *L_0_* = 5.1 mm.
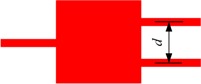 Multimode interference	Self-imaging effect [19]	When *d* = 50 μm, The minimum length of a single branch: *L_0_* > 12 mm.

### 4.2. Trench-Based Bend and Branch

**Figure 5:**
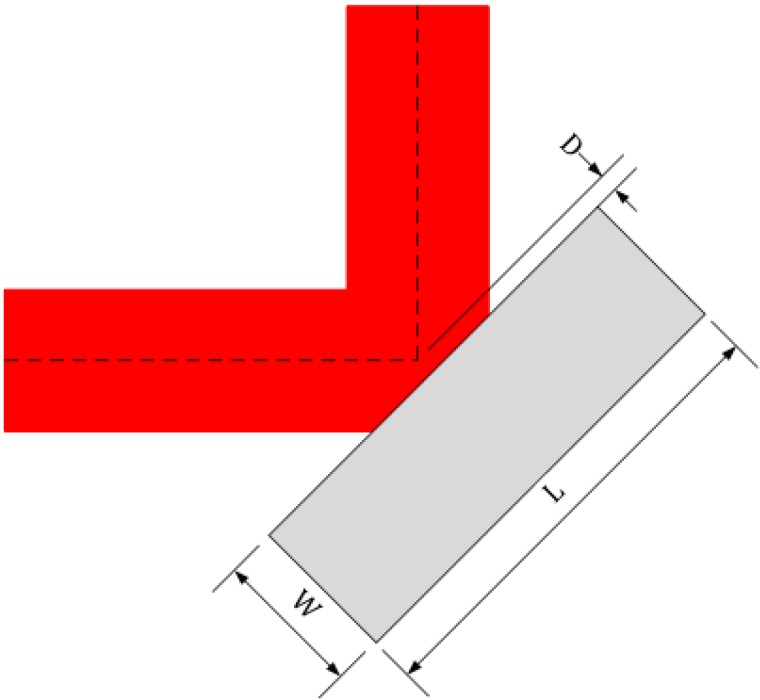
90° trench-based bend geometry of SOI rib waveguide with large cross section.

**Figure 6:**
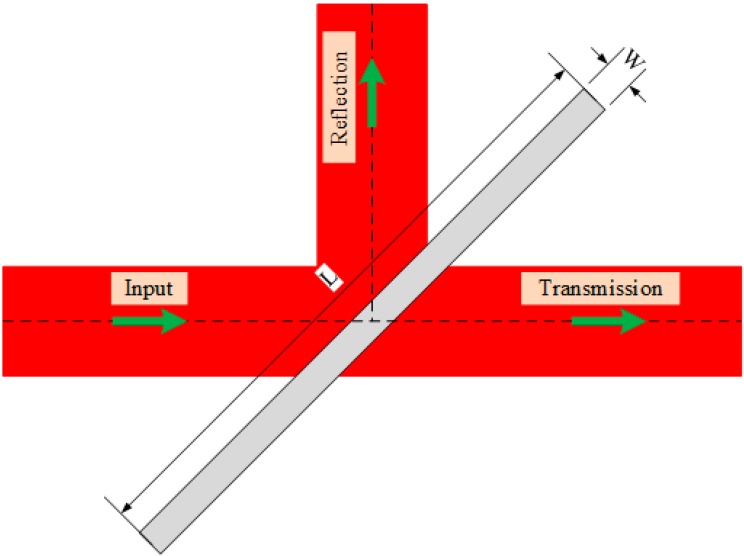
T-shaped branch geometry of SOI rib waveguide with large cross section.

Studies have shown that the bends and branches of waveguides can be realized by using the medium trench, slot, or photonic crystal [20,21,22,23,24]. In this paper, media-filled trenches are used to achieve waveguide bends and branches for an SOI rib waveguide with a large cross section, as shown in Figure 5 and Figure 6, respectively. The gray area in these figures represents the filling medium, which can be air, SU8 or other refractive index matching fluid.

Due to the large dimension of the SOI rib waveguide, the computational memory requirement and time-consumption of a three-dimensional finite difference-time domain (3D-FDTD) simulation are huge. The usual way to overcome this issue is to simplify the 3D structure to 2D by using effective refractive index method (EIM), and then simulate the interested structure using two-dimensional finite difference-time domain (2D-FDTD) [25]. In this paper, a 2D-FDTD method with a perfectly matched layer (PML) boundary condition [26] is employed to numerically simulate the above mentioned waveguide bends and branches.

**Figure 7:**
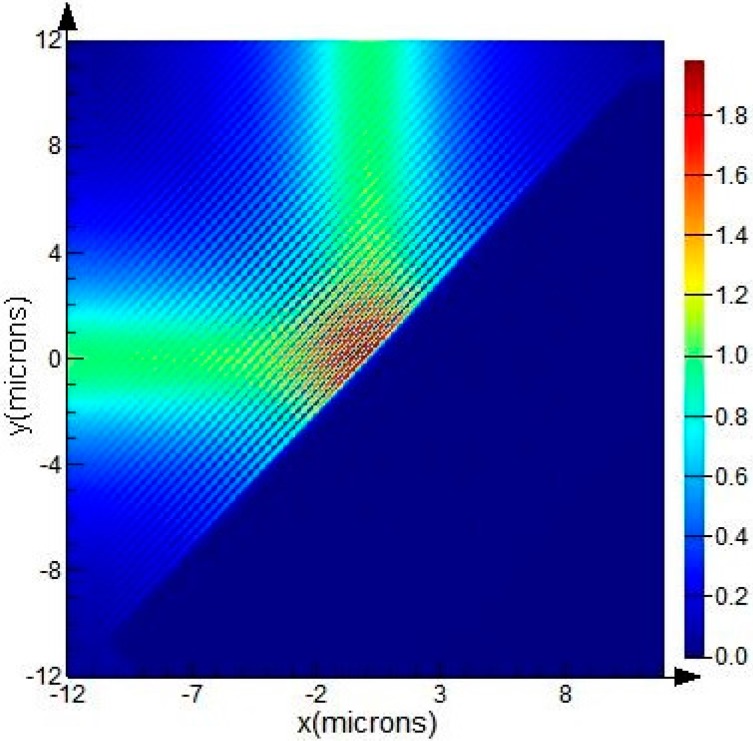
The electric intensity map in a plane 4 μm above the SiO_2_ layer for a 90° air-trench bend at a wavelength of 1550 nm. The SOI rib waveguide with a large cross section possesses a total rib height of 10 μm, an outside rib height of 5 μm, and a rib width of 2.5 μm. The guiding mode is the fundamental EH mode. The width and length of the air trench is 10 μm and 30 μm respectively, and the Goos-Hanchen shift compensation *D* = 0. Bend efficiency calculated through the 2D-FDTD simulation with mesh grid of 5 nm is 0.999746.

The medium trench shown in Figure 5 corresponds to a corner mirror, which can change the propagation direction of the waveguide according to total internal reflection. In general, the length and width of the medium trench (*L* and *W* as shown in Figure 5) should be large enough to reflect instead of to transmit more mode energy. The parameter *D* is defined to account for the Goos-Hanchen shift compensation, and it is positive when the trench interface moves away from the bending center (corresponding to the case shown in Figure 5, *D > 0*). When the trench interface precisely passes through the bending center, *D* = 0. Setting the width and length of the air trench at 10 μm and 30 μm respectively, *i.e. L* = 30 μm, *W* = 10 μm, the electric intensity map of a 90° air-trench bend is shown in Figure 7.

**Figure 8:**
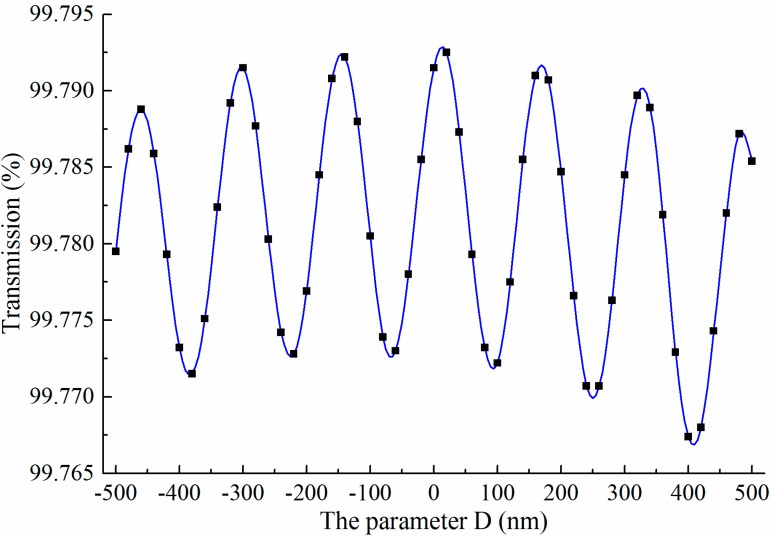
The bend efficiency as a function of the Goos-Hanchen shift compensation (parameter *D*). There is an oscillatory variation, but the amplitude is very small.

**Figure 9:**
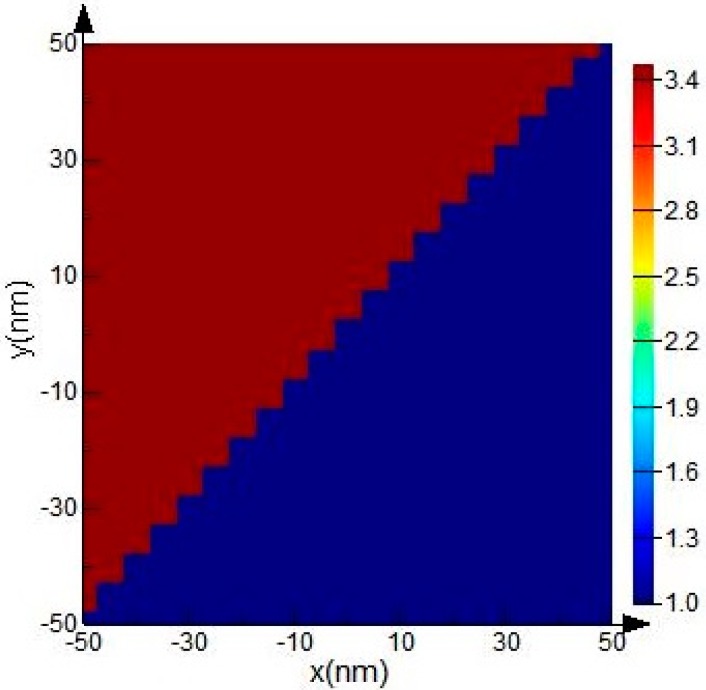
The local meshing in the 2D-FDTD simulation at the interface between air-trench and the bending waveguide. The mesh grid is 5 nm.

The influence of the parameter *D* on the bend efficiency of the air-trench bend exhibits as a curve with oscillatory variation in a very small range, as shown in Figure 8. Due to the strong ability of light constraint, there is a high bend efficiency for SOI rib waveguide with large cross section, even when *D* = ±500 nm. The reason for this oscillatory variation is that there is an angle between the air-trench and the meshing direction in 2D-FDTD simulation to avoid oblique incident light, and the interface of the air-trench and the waveguide is jagged as shown in Figure 9. It can be verified that this effect will be attenuated if finer mesh sizes are adopted.

**Figure 10:**
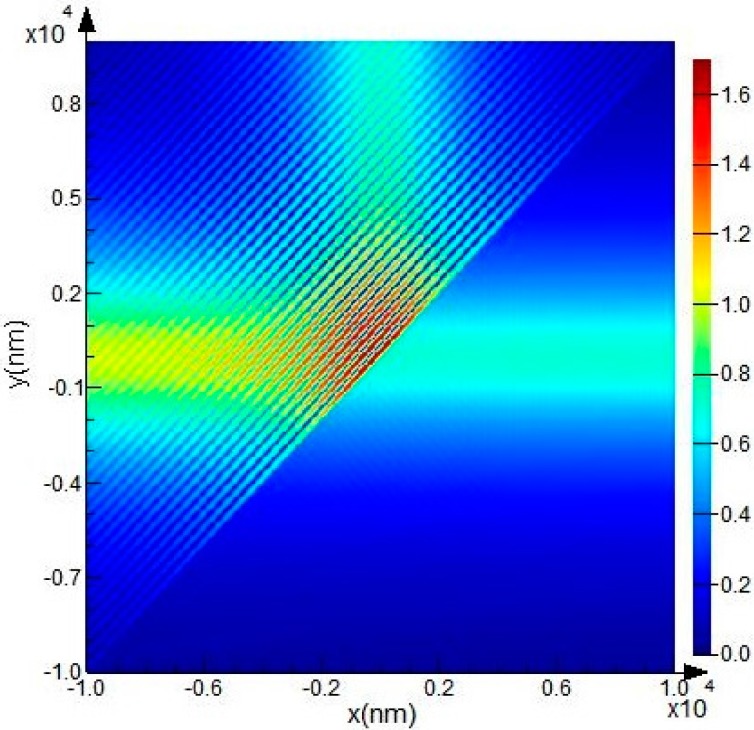
The electric intensity map in a plane 4 μm above the SiO_2_ layer for an asymmetric T-shaped air-trench branch at a wavelength of 1550 nm. The SOI rib waveguide with a large cross section possesses total rib height of 10 μm, outside rib height of 5 μm, and rib width of 2.5 μm. The guiding mode is the fundamental EH mode. The width and length of the air trench is 97 nm and 30 μm respectively.

Similarly, the electric intensity map of the asymmetric T-shaped air-trench branch with the trench width and length of 97 nm and 30 μm respectively is shown in Figure 10. Based on 2D-FDTD simulation with the mesh grid of 5 nm, the reflection efficiency is 0.497357 and the transmission efficiency is 0.502518.

**Figure 11:**
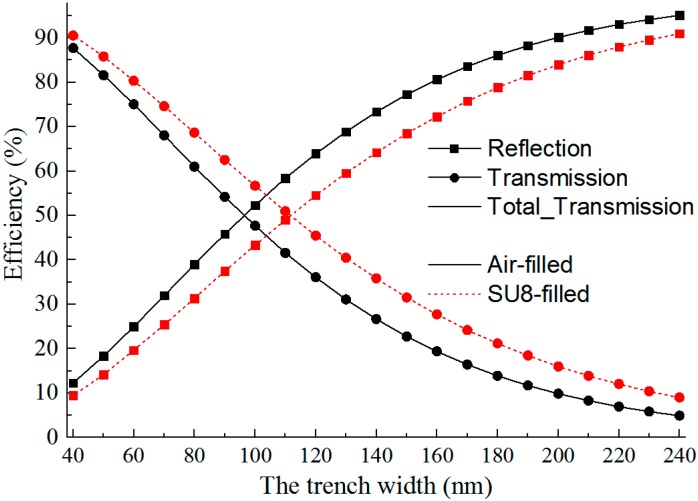
Splitting efficiency as a function of trench width. A 50%:50% splitting ratio is achieved with 96 nm air-trench width or 112 nm SU8-trench width.

It is found from the simulations that the splitting efficiencies, including the reflection efficiency, the transmission efficiency and the total transmission efficiency, are functions of trench width (w), as shown in Figure 11. Both for air-filled trench and SU8-filled trench, the transmission decreases and the reflection increases as the trench width increases. With the trench width of 96 nm for air-trench branch and 112 nm for SU8-trench branch, a splitting ratio of 50%:50% is achieved.

**Figure 12:**
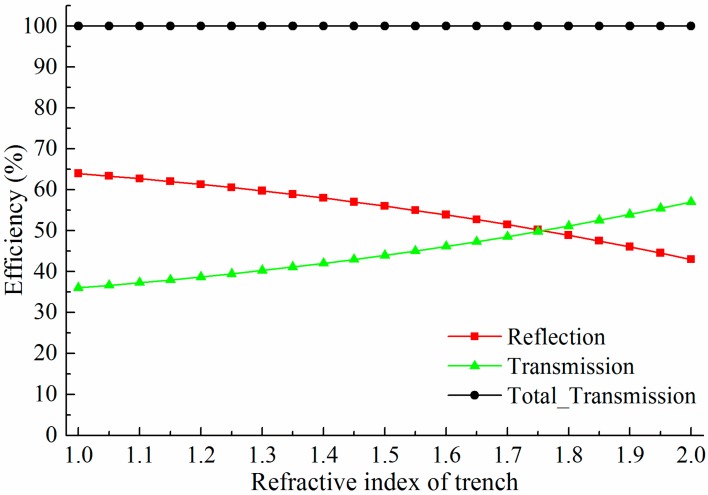
Splitter efficiency as a function of refractive index of trench. When the trench width is 0.12 μm, a 50%:50% splitting ratio is achieved with the refractive index of 1.76.

Supposing the trench branch is filled with index matching fluid and has a trench width of 120 nm, the influence of the refractive index of the index matching fluid is shown in Figure 12. As expected, the higher refractive the index of the filled material, the smaller the reflection efficiency and the larger the transmission efficiency. Thus, it can be concluded that the higher refractive index of the filled material results in the larger trench width for a 50%:50% splitting ratio. In addition, using index matching fluid as the filled material for the trench branch can prevent pollution of dust or impurities in the air.

Overall, the trench-based bends and branches can be used for SOI rib waveguide with large cross section, and their performances are obviously superior to the traditional bending and branching structures, such as the higher transmission efficiency and the shorter length of a single branch. Note that the simulation errors mainly come from two aspects, the coupling between modes and the out-of-plane scattering loss, in the process of simplifying the 3D model to 2D [27]. Because the SOI rib waveguides discussed in this paper are confined by the single-mode condition and have strong capability of light constraint, the influences caused by these two factors are very small, and thus the error of the above results obtained from 2D-FDTD simulations is insignificant.

### 4.3. Proposed MZI Structure

**Figure 13:**
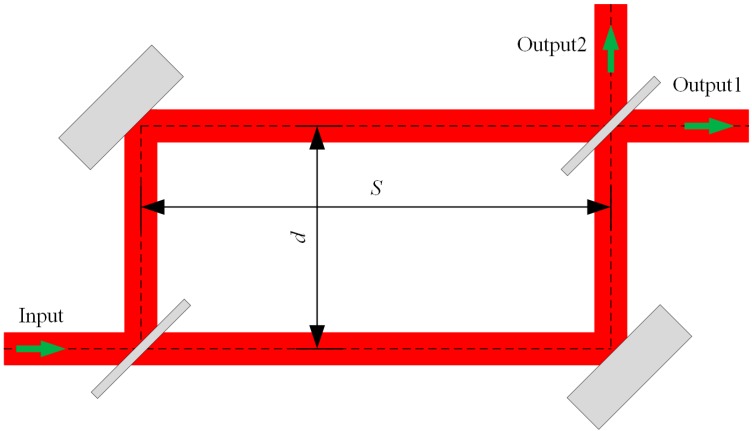
Schematic of the MZI using media trenches based on SOI rib waveguide with large cross section.

A new MZI structure based on an SOI rib waveguide with a large cross section that consists of two trench-based waveguide bends, two trench-based waveguide branches, and two straight waveguides is proposed as shown in Figure 13. These two parallel straight waveguides can act as the reference arm and the sensing arm of the MZI sensor, and their spacing is denoted by *d*. The parameter *S* represents the horizontal distance of the two waveguide branches. Compared with the traditional MZI configurations, the proposed configuration possesses two out ports, which can be used solely, or synchronously as mutual reference.

**Figure 14:**
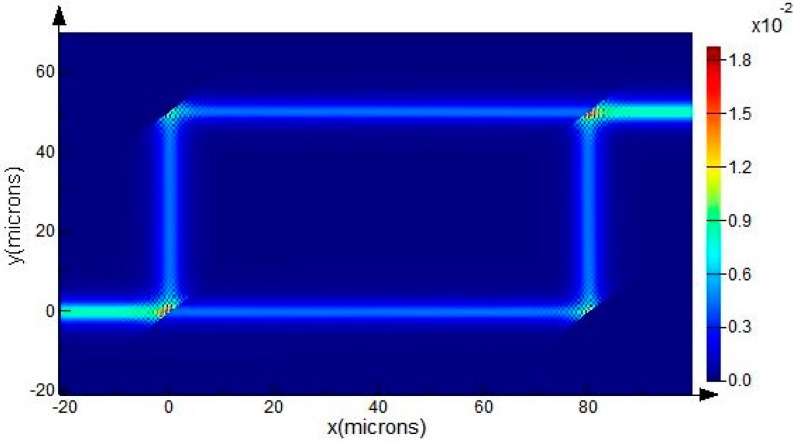
The optical power map of the MZI structure with the fundamental EH-polarized mode of an SOI rib waveguide with *H* = 10 μm, *h* = 5 μm, *w* = 2.5 μm at wavelength of 1550 nm. Because there is no phase difference between the sensing arm and the reference arm, only the port “output 1” has an output signal.

**Figure 15:**
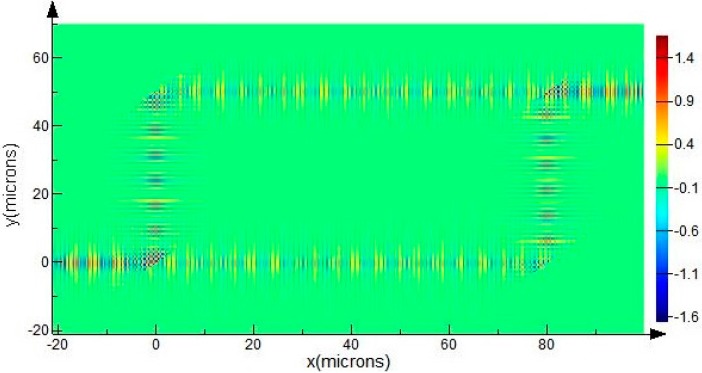
The transmission of the electric field components in Z direction Ez (out of plane) of the MZI structure with EH-polarized mode of SOI rib waveguide with *H* = 10 μm, *h* = 5 μm, *w* = 2.5 μm at wavelength of 1550 nm.

Using a 2D-FDTD simulation, the optical power map of such a trench-based MZI structure with *d* = 50 μm, *S* = 80 μm is shown in Figure 14. The guiding mode is the fundamental EH mode of the SOI rib waveguide with *H* = 10 μm, *h* = 5 μm, *w* =2.5 μm at wavelength of 1550 nm. It shows that only one output port has power output due to the interference effect of the two identical guiding modes in the sensing arm and the reference arm. The transmission of the electric field components in the Z direction *E_z_* (out of plane) can be used to clearly explain this interference case, as shown in Figure 15.

**Figure 16:**
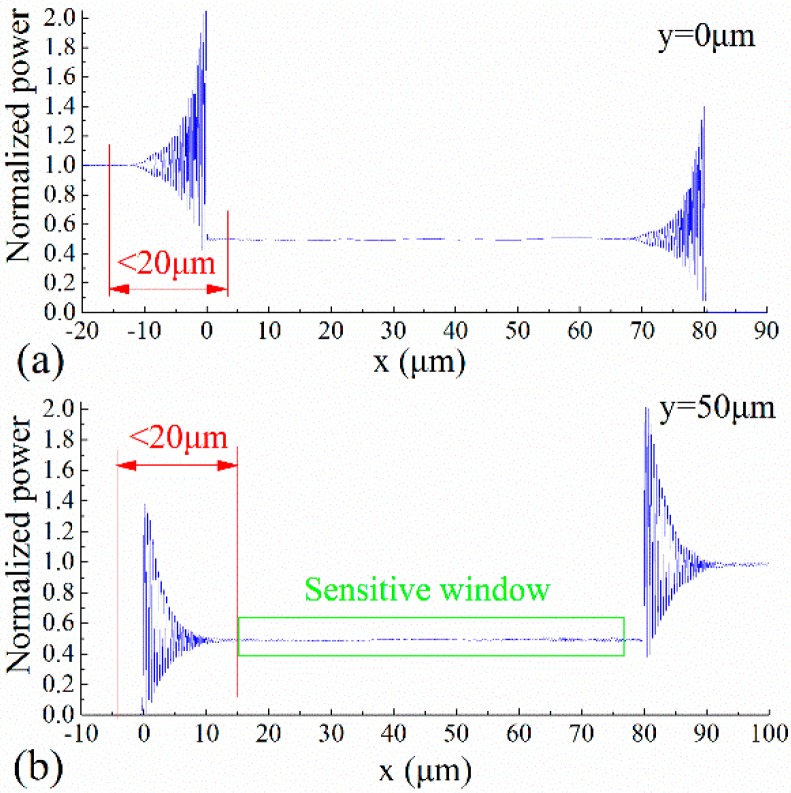
Normalized power of the SOI rib waveguide mode propagating along the sensing arm and the reference arm.

Figure 16a,b respectively show the normalized power of the SOI rib waveguide mode that propagates along the sensing arm and the reference arm. This normalized power is the ratio of the power on the transmission cross section to the input power of the light source. It can be seen that there are unstable regions at the waveguide bends and branches, which are caused by the interference of the waveguide modes. In particular, the normalized power of the interference enhancement is more than *1*. It is found that the length of the unstable transmission segments at the waveguide bends or branches are less than 20 μm, which is a great advantage compared to the conventional implementations of MZI.

**Figure 17:**
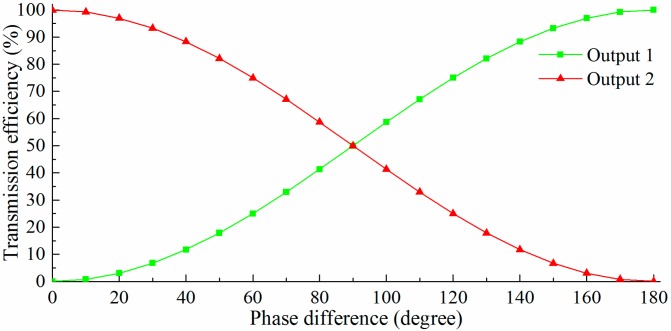
The normalized output power in each output port as a function of the phase difference between the sensing arm and the reference arm.

In the ideal situation (the splitting ratio of every branch in the MZI is 50%:50%), the normalized output power of each output port is a function of the phase difference (*ΔΦ*) between the sensing arm and the reference arm, as shown in Figure 17. A complementary relationship between the two output ports can be found. The normalized output powers of the output port “output 1” can be expressed as Equation (5). There, *ΔΦ_0_* is the initial phase difference caused by machining error or other unbalanced factor.

(5) $$P\text{=}\frac{1}{2}\left[1+\cos(\Delta \Phi +\Delta \Phi_{0})\right]$$

## 5. MZI Sensing Platform

**Figure 18:**
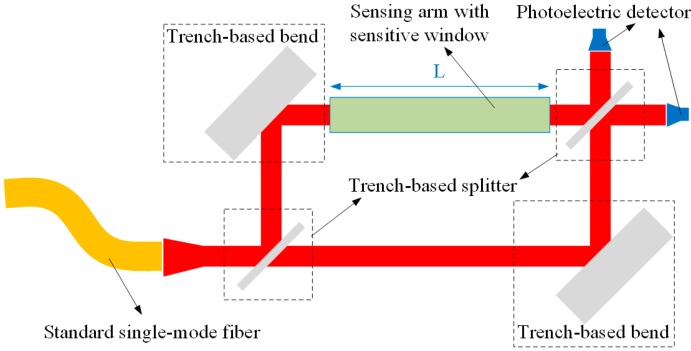
Schematic of the MZI sensing platform based on SOI rib waveguide with large cross section.

According to the above analyses and results, the trench-based MZI structure exhibits better performance than traditional configurations. The schematic of the MZI sensing platform based on SOI rib waveguide with large cross section is shown in Figure 18. There is a sensitive window at the sensing arm where selective biochemical sensitive material is used for a specific application. Due to the high coupling efficiency of the SOI rib waveguide with large cross section and the standard single-mode glass fiber, the input and output ports of the MZI sensing platform can be conveniently connected to a laser source and light power detection unit, and remote measurement based on fiber-optic communication can be achieved. With the help of a simple tapered mode converter, the butt coupling of input and output waveguides with standard single mode fiber can be easily realized. A set of simulations show that more than 80% of the coupling efficiency is very easy to achieve. In addition, the output signal can also be detected by integrated photoelectric detectors, as shown in Figure 18.

As an example, an MZI sensor with the fundamental EH-polarized mode of SOI rib waveguide with *H* = 10 μm, *h* = 5 μm, *w* = 2.5 μm at wavelength of 1550 nm, when *L* = 10 mm and *d* = 50 μm, a homogeneous sensitivity of 7296.6%/refractive index unit (RIU) can be obtained according to Equations (3) and (5). Supposing the resolutions of the photoelectric detectors connected to the output ports are 0.2% of the optical power for the intensity measurement, the MZI sensor can achieve a detection limit of 2.74 × 10^−6^ RIU. This detection limit of refractive index is smaller than that obtainable by Si_3_N_4_ rib waveguides (7 × 10^−6^ RIU) [28] and close to that reported in the previous works [29,30]. More importantly, employment of the SOI rib waveguides with large cross sections make the device match well with the communication glass fibers, and the size of the MZI's branches are very small, so that the entire sensing platform can be very compact.

## 6. Discussion

Employing with the media-filled trenches, the MZI configuration based on the SOI rib waveguide was realized in micrometer scale, thus a micron-sized and compact biochemical sensing platform based on an SOI rib waveguide with a large cross section was obtained, which is the main advantage of this work. Moreover, high coupling efficiency with standard single-mode glass optical fiber is the most important advantage of SOI rib waveguide with large cross section, which enables the waveguide sensors to integrate with optical fiber communication systems and (opto-) electronic systems, and therefore to realize remote sensing, *in situ* real-time detecting and possible application in the internet of things.

According to the above analysis, the MZI sensor can perform bulk sensing with high sensitivity, which can be used to as chemical sensor. Due to the simple MZI configuration and strong adaptability of evanescent field sensing, the MZI sensing platform can also be used to detect biological reactions as long as suitable receptor molecules are immobilized at the waveguide surface in the sensitive window.

To achieve high sensitivity, the trenches of the waveguide branches in the MZI configuration needs to be very narrow, which at the present stage may involve expensive microfabrication processes such as electron beam lithography (EBL) and deep reactive ion etching (DRIE). Therefore a compromise should be made between the sensitivity and the cost of the process. Alternately, employment of filled medium with higher refractive index can lower the difficulty of the process without decreasing the sensitivity. From the analysis of this paper, it can be speculated that the surface roughness of processing and the change of the external environmental temperature in real measurement have effects on the splitting efficiencies of the MZI’s branches, but the further effects on the performance of the sensing system need to be further researched in the experiments, which is the next step of this work. In addition, due to the strong anti-interference capability of MZI configuration, these effects might be very small. Fortunately, due to the mass production feature of the SOI process, a very low cost for each sensor chip can be achieved as long as a large market is found.

## 7. Conclusions

An MZI biochemical sensing platform based on an SOI rib waveguide with a large cross section is proposed in this paper. The optimization of the cross section dimensions of the SOI rib waveguide is performed through FDM simulations with the target of maximizing the evanescent field intensity. Medium filled trenches are employed to realize the MZI configuration based on the SOI rib waveguide. The performances of the MZI sensing platform are simulated by using 2D-FDTD method.

The optimization of the SOI rib waveguide is that the guiding mode is the fundamental EH mode, and the cross section dimension is that the total rib height *H* = 10 μm, the outside rib height *h* = 5 μm and the rib width *w* = 2.5 μm. When the length of the sensitive window *L* = 10 mm and the spacing distance between the sensing arm and the reference arm *d* = 50 μm, the MZI sensor based on SOI rib waveguide with large cross section at an operating wavelength of 1550 nm can achieve a homogeneous sensitivity of 7296.6%/RIU. Supposing the resolutions of the photoelectric detectors connected to the output ports are 0.2%, the MZI sensor can achieve a detection limit of 2.74 × 10^−6^ RIU.
